# Anti-Oxidant and Anti-Inflammatory Activity of Ketogenic Diet: New Perspectives for Neuroprotection in Alzheimer’s Disease

**DOI:** 10.3390/antiox7050063

**Published:** 2018-04-28

**Authors:** Alessandro Pinto, Alessio Bonucci, Elisa Maggi, Mariangela Corsi, Rita Businaro

**Affiliations:** 1 Department of Experimental Medicine, Sapienza University of Rome, 00161 Roma, Italy; 2 Department of Medico-Surgical Sciences and Biotechnologies, Sapienza University of Rome, 04100 Latina, Italy; bonucci.alessio@libero.it (A.B.); elisa.maggi@biologo.onb.it (E.M.); mariangela.corsi@uniroma1.it (M.C.); rita.businaro@uniroma1.it (R.B.)

**Keywords:** Ketogenic diet, Alzheimer’s disease, neuroprotection, inflammation, oxidative stress

## Abstract

The ketogenic diet, originally developed for the treatment of epilepsy in non-responder children, is spreading to be used in the treatment of many diseases, including Alzheimer’s disease. The main activity of the ketogenic diet has been related to improved mitochondrial function and decreased oxidative stress. B-Hydroxybutyrate, the most studied ketone body, has been shown to reduce the production of reactive oxygen species (ROS), improving mitochondrial respiration: it stimulates the cellular endogenous antioxidant system with the activation of nuclear factor erythroid-derived 2-related factor 2 (Nrf2), it modulates the ratio between the oxidized and reduced forms of nicotinamide adenine dinucleotide (NAD^+^/NADH) and it increases the efficiency of electron transport chain through the expression of uncoupling proteins. Furthermore, the ketogenic diet performs anti-inflammatory activity by inhibiting nuclear factor kappa-light-chain-enhancer of activated B cells (NF-kB) activation and nucleotide-binding domain, leucine-rich-containing family, pyrin domain-containing-3 (NLRP3) inflammasome as well as inhibiting histone deacetylases (HDACs), improving memory encoding. The underlying mechanisms and the perspectives for the treatment of Alzheimer’s disease are discussed.

## 1. Introduction

According to the World Alzheimer Report 2016, there are 47 million people worldwide suffering from dementia, and this number will rise to 131 million by 2050 due to population aging. This data also implies a great economic impact, which is expected to be one billion dollars worldwide by 2018. Despite of recent progress in understanding the neurobiology and pathophysiology of Alzheimer’s disease (AD), no resolutive pharmacological treatments but only a few symptomatic drugs are currently available to patients. Based on this consideration, much effort is aimed at the development of effective prevention lines: so far many non-pharmacological treatments have been proposed, including lifestyle interventions such as targeted nutritional protocols, caloric restriction and exercise, as well as mental challenges and socialization [1]. 

Moreover, although the effects of diet in aging and neurodegeneration is becoming well-established, the evidence regarding the effect of diet on cognition in neurological disorders has not been proved yet. This is surprising because preservation of cognition is often a predominant concern for patients with neurological disorders, perhaps due to the substantial impact on occupation, social relationships, activities of daily living, and quality of life [2].

Alzheimer’s disease etiopathogenesis has been linked to oxidative stress, neuroinflammation, mitochondrial impairment, hypometabolism and blood-brain barrier (BBB) disruption [3,4,5,6].

The proved effectiveness of the ketogenic diet in childhood refractory epilepsy and the early metabolic dysfunction observed in mild cognitive impairment (MCI), which usually precedes the onset of AD, paved the way to evaluate the possible therapeutic role of dietary metabolic approaches to AD and other neurodegenerative diseases. The brain hypometabolism was associated to a reduced glucose utilization, detected by fluorodeoxyglucose positron emission tomography (FDG-PET), but several alteration in amino acids and lipids metabolic pathways have been recently identified through modern metabolomics techniques [7,8,9,10,11]. 

Chronic inflammation and oxidative stress are considered two key factors in the development of Alzheimer’s disease, underlying neurotoxic mechanisms leading to neuronal death occurring in the brain areas responsible for memory and cognitive processes [12]. A bulk of studies have shown that the blood-brain barrier is impaired in Alzheimer’s disease resulting in an altered expression of some transporters, including the down-regulation of glucose transporter. The primary fuel for the brain is glucose that must be taken from the blood and transported across the BBB by the specific glucose transporters (GLUTs) due to the inability of neurons to synthesize or store it. When there is a decreased expression of these transporters, as it has been shown in AD, Ketone Bodies (KBs) become the alternative energy source to glucose for the brain due to their ability to cross the BBB carried by specific transporters that are not down-regulated during AD. For all these reasons many studies have been performed to verify if the induction of a mild ketosis may favor the trophism of neuronal cells in the course of AD.

In the last few years the number of publications about the ketogenic diet (KD) and neurological disease have been progressively increasing. The beneficial effects of these diets for enhancing cellular metabolism and mitochondrial function, inducing a shift in energy metabolism, have been described in more detail, although the specific effects on cognition have not been analyzed in such depth [13,14]. The aim of this review is to summarize the current evidence regarding KD and AD with specific reference to antioxidant and anti-inflammatory mechanisms, as well as their effects on cognitive processes. 

## 2. Ketone Bodies as Alternative Fuel for the Brain

The adult brain represents about 2% of body weight, but it consumes about 20–23% of whole-body energy expenditure, mostly in the form of glucose. Indeed, during episodic period of starvation or low-carbohydrates intake, the KBs metabolism becomes the main source of alternative energy as a results of carbohydrate deficiency and increased availability of fatty acids from lipolysis [15]. β-hydroxybutyrate (βHB) and acetoacetate (AcAc) can replace glucose in the brain, whereas acetone, produced by spontaneous decarboxylation of AcAc, is rapidly eliminated through urine and lungs. Under human physiological condition plasma KBs concentration fluctuate between 100 and 250 mM, increased to ~1 mM after prolonged exercise or 24 h of fasting and to 6–8 mM during a prolonged fast without giving rise to clinically hazardous acidosis. In diabetic ketoacidosis, plasma concentration of KBs can exceed 20 mM [16,17]. The human liver produces up to 300 g of ketone bodies per day which contribute between 5% and 20% of total energy expenditure in fed, fasted, and starved states [15].

## 3. Ketogenic Diets 

The classic KD was designed in 1923 for the treatment of epilepsy by Dr. Russell Wilder at the Mayo Clinic. Ketogenic diets are composed of high amount of fat and low carbohydrates, typically at a macronutrient ratio of fat to protein plus carbs equal to 3–4:1 (4 grams of fat to 1 gram of protein and carbohydrate combined) which approximately results in 90% of total calories intake from fat, 6% from protein, and 4% from carb (because fat has 9 calories per gram, while both protein and carb have just 4 calories per gram). The moderate protein intake avoids amino acids induced gluconeogenesis and promotes KBs formation. The resulting metabolic profile is characterized by a slight reduction of blood glucose concentration with increasing KBs and mimics the fasting state with the brain beginning to use KBs to generate cellular energy. The very low carbohydrate intake of classic KD is difficult to maintain and consequently at least five main variants of the KD were published in medical literature as treatments for diseases that have an underlying metabolic dysregulation, such as epilepsy, cancer, and Alzheimer’s disease. All KDs are a variation of the classic KD, the main difference being the macronutrient ratio: the Modified Ketogenic Diet (2:1–1:1), the Modified Atkins Diet (MAD; 1:1), and more recently the Medium-Chain Triglyceride (MCT) oil diet (1.9:1), Low Glycemic Index Treatment (LGIT) and Intermittent Fasting [2,6]*.* Usually, fat type and chain length are not specified, but typically a ketogenic diet includes mostly saturated fats, which have 16–20 carbon atoms. In the last few years the alternative medium-chain triglyceride (MCT) ketogenic diet was developed, comprising about 60% octanoic acid (an eight-carbon fatty acid) and about 40% decanoic acid (a ten-carbon fatty acid). The quick metabolism of the shorter fatty acids results in more efficient generation of ketone bodies allowing to assume a greater proportion of carbohydrates with only about 45% of lipid energy intake [2,6].

A key issue is the potential occurrence of adverse effects in course of KD treatment. The incidence of adverse effects is primarily associated with the KD model applied, with the classic KD (4:1 ratio) burdened by a lower tolerance when compared with the 3:1 ratio KD or MAD, and it is significantly reduced by the current availability of specific dietary supplements or formulas. The most common adverse events are the gastrointestinal symptoms, including vomiting, constipation and diarrhea; moreover, weight loss and hyperlipidemia are potential side effects whereas the major adverse events (dehydration, electrolyte alteration, arrythmias) are uncommon. During the first few days and weeks after KD initiation, mild side effects such as headaches, fatigue, can occur. The pathological ketoacidosis due to a lack of insulin in type 1 diabetes is not possible as result of dietary changes alone. The frequency evaluation of KD side effects in patients with AD is difficult due to the small number of available clinical trials and the heterogeneity of KD model applied [18,19,20,21].

Although several clinical trials were carried out on childhood refractory epilepsy, adverse events have not been consistently reported [22]. As well as children, elderly people subjected to long-term KD treatment require a close monitoring for adverse events by a neurologist, nutritionist and dietitian, and they should be subjected to a periodic dual energy X-ray absorptiometry (DEXA) screening for body composition health (bone, fat free mass, muscle mass and fat mass). 

## 4. Mechanisms Underlying the Beneficial Effects of Ketogenic Diet on Neurological 

Despite the poor number of clinical trials experimenting on the effectiveness of different types of ketogenic diets on Alzheimer’s cognitive impairment (summarized in Table 1), a growing body of biological mechanisms potentially able to explain the observed clinical effects have been proven in in vitro or in animal model studies.

The therapy of AD was primarily addressed to the prevention of the specific histological injures (amyloid plaques, neurofibrillary tangles) but the lack of success moved the focus of research to functional dysregulation of brain metabolism, mitochondrial homeostasis and excitatory and inhibitory neuronal signaling, laying the foundation for the development of a “metabolic therapy”.

Before describing more extensively the activity of ketogenic diet associated to the decrease of the oxidative damage and to the modulation of inflammatory status, the main mechanisms reported in the scientific literature are briefly summarized. 

The putative neuroprotective effects of ketone bodies have been associated with the following mechanisms:

- increasing intracellular adenosine triphosphate (ATP) availability;
- reducing ROS generation by mitochondrial complex I;
- inhibiting mitochondrial permeability transition;
- stimulating mitochondrial biogenesis, resulting in stabilized synaptic function;
- altering metabolism of neurotransmitters such as glutamate and gamma-amino butyric acid (GABA);
- activating energy-sensing signaling pathways such as the peroxisome proliferator activated receptor (PPAR), mammalian target of rapamycin (mTOR), and adenosine monophosphate- (AMP)-activated kinase (AMPK) pathways.

The advances in understanding of the mechanisms of action of medium-chain fatty acids (e.g., decanoic acid and octanoic acid) have more recently shifted attention away from ketone bodies to the direct role of fatty acids as a therapeutic effectors, paving the way for novel dietary and drug therapies for epilepsy and other disorders, as comprehensively described in Augustin’s recent review. According to this review medium-chain fatty acids are able to cross the blood–brain barrier reaching in the brain a concentrations more than 50% greater than the plasma one, providing an alternative energy source for brain neurons and astrocytes. Octanoic acid seems to undergo β-oxidation in astrocytes more easily than decanoic acid and readily produces ketone bodies; decanoic acid instead stimulates glycolysis producing lactate available as fuel for the brain cells [6].

## 5. The Antioxidant Activity of the Ketogenic Diet: An Insight into Molecular Mechanism 

Mitochondria are the main source of energy in the cell, implicated in ATP production through oxidative phosphorylation. They play a key role in apoptosis and production of reactive oxygen species (ROS) as a result of escape of electrons during the transfer along the electron transport chain (ETC). Accumulation of ROS, primarily derived from mitochondrial complexes I and III [30,31], damages proteins, lipids and nucleic acids. Oxidative stress causes mitochondrial dysfunction which enhances ROS production in a pathological positive feedback loop. 

KBs metabolism reduces oxidative stress compared to glycolysis, hypothesizing a further neuroprotective mechanism carried out by these compounds [32].

β-Hydroxybutyrate (β-HOB), the most studied KB, has been shown to reduce the production of ROS improving mitochondrial respiration and bypassing the complex I dysfunction [33,34].

**(a)**  The ketogenic diet (KD) stimulates the cellular endogenous antioxidant system with the activation of nuclear factor erythroid-derived 2 (NF-E2)-related factor 2 (Nrf2), the major inducer of detoxification genes.

Nrf2 is a member of the cap “n” collar (Cnc) family of transcription factors, a 605 amino acid protein, with six functional domains termed Nrf2-erythroid-derived cap’n’collar homolog (ECH) homologues (Neh). Under homeostatic conditions, Nrf2 is sequestered and inhibited in the cytoplasm by kelch-like ECH associated protein 1 (Keap1) interacting with Neh2 domains of Nrf2 [35,36]. Keap 1 is a substrate adaptor for different ubiquitin ligase systems, Cullin 3 (Cul3) RING-box 1 (RBX1) E3 ubiquitin ligase complex being the most studied [37]. They constantly ubiquitinate Nrf2 which is then degraded by the proteasome [38,39]. 

Nrf2-Keap 1 can be considered a sensor of cellular redox status since Keap 1 is a cysteine-rich protein. When distinct cysteine residues (Cys273 and Cys288) are oxidized, a conformational change takes place in Keap1 and so Nrf2 is able to translocate to the nucleus [40,41]. The translocation of Nrf2 is also possible if protein kinase C δ (PKCδ) phosphorylates Ser40 on Nrf2 and if Cys151 on Keap1 gets oxidized [42]. 

Once into the nucleus Nrf2 constitutes a heterodimer with small Mafs [43] and binds to the antioxidant responsive element (ARE) [44,45]. ARE is an enhancer element in the promoter region of cytoprotective genes such as proteins with thiol (-SH) group like glutathione (GSH), thioredoxin (TXN) and piroxiredoxin (PRDN) or detoxification enzymes like superoxide dismutase (SOD), catalase, heme oxygenase-1 (HO-1) and glutamate cysteine ligase (CGL) ([46,47,48]. Nrf2 is able to induce glutathione reductase, thioredoxin and peroxiredoxin, fundamental enzymes implicated in the regeneration of active form of endogenous antioxidants [49]. 

Moreover, β-HB is an endogenous inhibitor of class I and class IIa histone deacetylases (HDACs). The inhibition of HDACs (1–5,7,8,9) upregulates the transcription of detoxifying genes including catalase, mitochondrial superoxide dismutase (mn-SOD) and metallothionein 2 in order to counteract oxidative stress [50,51].

Hippocampus of rats fed a KD for up 3 week showed Nrf2 activation and nuclear translocation [52,53]. CGL subunits (GCLC and CGLM) have antioxidant-response elements (ARE)-like sequences: in this work CGL biosynthesis increases as well as glutathione but there is a decrease in the mitochondrial level of ROS. How KD activates the Nrf2-ARE pathway is not completely understood, Milder et al. [53] have showed that in hippocampal mitochondria of KD-fed rats there is an acute increase of H_2_O_2_ level after only 1 day. The first boost of H_2_O_2_ production is in contrast with the reduction of its level after 3 week of diet. Probably H_2_O_2_ acts as a redox signal which initiates signaling cascade. Interestingly H_2_O_2_ enhances DNA binding of Nrf2 to the ARE [54]. Nrf2 activation is assisted by lipid peroxidation: for example arachidonic and linolenic acid during mitochondrial dysfunction may react with reactive nitrogen species (RNS) and ROS to produce 4-hydroxy-2-nonenal (4-HNE), an α, β-unsaturated aldehyde. Acute increase of H_2_O_2_ and 4-HNE during the first days of diet activates the release of Nrf2 from Keap 1 to produce an adaptive antioxidant response [52,55].

**(b)**  A further mechanism explaining the protective activity of the ketogenic diets against oxidative stress is the intracellular modulation of the NAD^+^/NADH ratio. An increased NAD^+^/NADH ratio protects against ROS and plays an important role in cellular respiration, mitochondrial biogenesis and redox reactions [56]. Nicotinamide adenine dinucleotide (NAD) presents two forms: the oxidized form (NAD^+^) and the reduced form (NADH). During the glycolytic pathway glucose reduces 4 molecules of NAD^+^, while, on the contrary, the complete mitochondrial oxidation of β-HOB and acetoacetate (AcAc) reduces respectively 1 and none molecule of NAD^+^ [57]. In hippocampus and cerebral cortex of KD-fed rats a significant increase in the NAD^+^/NADH ratio was detected not only after 3 weeks but also after 2 days [58]. The fully oxidation of KBs and its product ATP inhibits glycolytic pathway which leads to a further accumulation of NAD^+^. A high NAD^+^/NADH ratio may influence gene expression through Sirtuin 1 (SIRT1), a type 3 histone deacetylase [59]. SIRT1 is a 747 amino acid NAD^+^ dependent enzyme with a globular highly conserved catalytic domain [60], it also possess a nuclear export signals and may translocate between the nucleus and the cytoplasm [61]. Tumor suppressor hypermethylated in cancer 1 (HIC1) together with C term binding protein (CtBP) can suppress SIRT1 gene expression. This suppressor complex dissociates when the glycolytic pathway is inhibited and NAD^+^/NADH ratio heightens (interestingly similar condition occurs during KD). When it happens, SIRT1 protein levels increase [62]. Even DNA damage, forkhead transcription factor (FOXO3A) [63], starvation with the cAMP response-element-binding protein (CREB) activation [64] might enhance the transcription of SIRT1. SIRT1 is implicated in many biological processes deacetylating histone and non-histone targets [65] and many of them seem to be associated with antioxidant properties of KD. Additionally, SIRT1 might exercise other functions to limit oxidative stress, such as improving synthesis of heat shock proteins, increasing antioxidant defenses, promoting DNA repairing activity of p53 and FOXO factors [66,67] and deacetylating Nrf2 [68].
**(c)**  Ketone metabolism increases the efficiency of electron transport chain (ETC) through the expression of uncoupling proteins (UCP) [69]. UCP reduces the mitochondrial membrane potential and diminishes the production of ROS and reactive oxygen nitrogen species (RONS) [70,71]. UCP4 and UCP5 are increased in brains of ketone ester-supplemented rats [72]. The KD inductive effect on UCP2, 4 and 5 expression could be a consequence of SIRT1 activation [73].
**(d)**  SIRT1 activation, mediated by KD, could offset mitochondrial dysfunction enhancing mitochondrial biogenesis [74]. In the cell there is a constant balance between the mitophagy of damaged mitochondria and the biogenesis to renew mitochondria population [75]. The major regulator of the biogenesis process is the Peroxisome proliferator-activated receptor Gamma Coactivator-1α (PCG-1α) whose transcription is triggered by Nrf2 [76,77]. SIRT1 plays a key role in deacetylase PCG-1α [78] which co-activates the transcription of Nuclear Respiratory Factor (NRF). NRF 1 and NRF 2 lead to the transcription of mitochondrial transcription factor A (TFAM), subsequently TFAM drives the replication of mitochondrial DNA and the mitochondrial biogenesis [79].
**(e)**  At last, ROS and reactive nitrogen species (RNS) can also open the mitochondrial permeability transition pore (mPTP). mPTP releases cytochrome c (Cytc) and apoptosis inducing factor (AIF) which activate caspases 3 and 9 that finally initiate the apoptotic pathway [80,81,82]. Mitochondrial ATP-sensitive potassium channels (mitoKATP channels) in the inner mitochondrial membrane are implicated in modulation of ETC and calcium buffering through enhancing K+ efflux [83]. During oxidative stress KBs can activate mitoKATP channels, improving the efficiency of ETC [84] and inhibiting mPT [85,86,87].

## 6. The Anti-Inflammatory Activity of Ketogenic Diet: An Insight into Molecular Mechanism

Several neurodegenerative diseases are interconnected by shared neurotoxic mechanisms related to inflammatory mechanisms such as cytokine release, neurotrophin synthesis inhibition, release of NO and CO, which are potentially harmful to surrounding tissues, and so on. Against this the cells provide endogenous mechanisms tending to neutralize the molecules responsible for inflammatory damage [12].

A winning strategy to attempt to block neuroinflammation may consist in the enhancement of endogenous anti-inflammatory programs. One of the most promising approaches seems to be the ketogenic diet that was set up in the first decade of the last century to treat refractory epilepsy and then applied with success to treat other neurological diseases [88]. 

A great interest has been focusing on the use of KD in the treatment of AD for two main reasons: (i) KD was shown to decrease the production of Amyloid Precursor Protein (APP) and therefore the βamyloid peptide; and (ii) KD was related to the activation of peroxisome proliferator-activated receptor gamma (PPARγ) and then to the decrease of systemic inflammation [89,90]. 

βHB, one of the main KB detected in the blood after the supply of a ketogenic diet, is able to cross the BBB thanks to the specific expression of its transporter MCT1 on endothelial cells. But after its entrance into the brain, β-hydroxybutyrate (βHB) not only provides energy but activates the receptor hydroxy-carboxylic acid receptor 2 (HCA2), lowering neuroinflammation [91].

HCA2, a G-protein coupled receptor, is expressed on macrophages, dendritic cells and microglia [92]. βHB which is the main endogenous ligand of HCA2, activates human HCA2 with an half maximal effective concentration (EC50) of about 700 mM, a concentration attained only after feeding a ketogenic diet [91]. HCA2 may elicit anti-inflammatory effects through the inhibition of NF-kB activation [93]. HCA2 activation induces a neuroprotective macrophage phenotype related to Prostaglandin D2 (PGD2) production by Cyclooxygenase 1 (COX1), that helps to resolve inflammation [94] and has neuroprotective effects [95]. A metabolite of PGD2 inhibits the main activator of NF-kB which is IKB kinase (IKK). As a consequence HCA2 activation inhibits NF-kB in macrophages [93]. The activation of HCA2 by ketogenic diet was shown to induce a neuroprotective phenotype in bone-marrow derived macrophages that infiltrate the brain [92]. 

HDACs are a group of proteins that are able to regulate gene expression by deacetylating lysine residues on histone proteins. It is known that hyperacetylation of histones is associated with the activation of gene expression and therefore HDACs with their action on histone proteins may suppress gene expression. Some studies in animal models have highlighted the role of HDACs inhibitors in enhancing mnesic processes suggesting their potential use in the treatment of cognitive diseases.

HDAC plays an important role in the pathogenesis of AD by altering chromatin structure and accessibility [96]. βHB inhibits HDACs 1, 3 and 4 (class I and IIa) in vitro [97]. Reducing HDAC2 in a mouse model for amyloid deposition via RNA interference was able to improve memory function and synaptic plasticity [98]. HDAC3 negatively regulates spatial memory in APP/Presenilin 1 (PS1) mice and HDAC3 inhibition might represent a potential therapy for the treatment of AD [99]. Reducing HDAC6 levels is mechanistically linked to tubulin-acetylation and improved mitochondria transport [100]. 

βHB mediated the inhibition of NLRP3 inflammasome in lipopolysaccharides (LPS)-stimulated human monocytes leading to a reduced production of interleukin-1beta (IL-1beta) and interleukin-18 (IL-18) without significantly affecting tumor necrosis factor-alpha (TNF-alpha) levels [101]. βHB dose-dependently inhibited IL-1beta and IL-18 secretion blocking NLRP3 inflammasome activation by limiting K^+^ efflux from cells [101]. Several experiments have ruled out a hypothetic role for mitochondrial ROS in the effects of ketone bodies on the inflammasome.

Since 2008 it has become clear that a low carbohydrate diet causes an increase of KB serum levels, paralleled by a reduction in several inflammatory parameters [102], and that KD allows neuroprotection for brain injuries and neurodegenerative diseases [103].

A randomized controlled dietary intervention trial was done on 40 overweight subjects aged 18–55 years, fed with a diet very low in carbohydrate or an isocaloric diet low in fat for 12 weeks. Subjects fed with KD developed a mild ketosis and showed weight loss, decreased adiposity, and an improved glycemic control as well as an improved insulin sensitivity. Both diets significantly decreased the concentration of several serum inflammatory markers, but there was an overall greater anti-inflammatory effect associated with KD: a larger reduction was observed in TNF-alpha, interleukin-8 (IL-8), monocyte chemoattractant protein (MCP-1), plasminogen activator inhibitor-1 (PAI-1), E-selectin, intercellular adhesion molecule-1 (ICAM-1), while these markers showed a little change in the subjects fed with a low fat diet, suggesting that it is the macronutrient composition, not the weight loss or the caloric reduction the real responsible for the anti-inflammatory activity [102].

Basic research provides evidence that KD play a neuroprotective role in some acute or chronic neurodegenerative diseases. Rats fed a KD for 3–4 weeks showed a reduced peripheral inflammatory response after the injection of Freund’s complete adjuvant in their paw, compared to animal fed a standard diet. Ketone metabolism results in decreased production of reactive oxygen species, known to contribute to inflammation. Moreover polyunsaturated polyunsaturated fatty acids activate PPAR which inhibit NF-kB and AP-1 [104].

KD neuroprotective activity has been analyzed previously, based on its anti-inflammatory effects.

In a model of experimental autoimmune encephalomyelitis KD reverted the increased expression of inflammatory cytokines, as well as the production of reactive oxygene species [105].

In a mouse model of Parkinson’s disease, where substantia nigra dopaminergic neurons dysfunction is induced by mPTP treatment, KD was shown to inhibit the proinflammatory activation induced by mPTP [106]. Activated microglia produce relevant proinflammatory citokines, including IL-1beta, IL-6, TNF-alpha which were associated to dying or damaged dopaminergic neurons [107]. In the study by Yang et al. [106] KD was able to decrease the activation of microglia and its products IL-1beta, IL-6, TNF-alpha, sparing at the same time tyrosine hydroxylase (TH)-positive neurons in substantia nigra, compared to control animals fed a standard diet. Moreover, the deficit of motor function induced by mPTP was partially but significantly recovered by KD pre-treatment. A study focused on Parkinson’s disease patients showed that a KD supplied for 28 days induced a significant improvement in symptoms [108].

## 7. Conclusions

The results obtained so far by applying the ketogenic diet to the treatment of several neurological diseases seem to be particularly interesting for the recovery of cognitive functions, although they are numerically limited. Different studies conducted on animal models have proposed a causal role for KD, although they do not always reflect with precision the pathogenesis underlying the development of neurological diseases. The few studies conducted on humans available so far are based on a pre/post design but without a reference control group and without randomization. Of particular interest were the RCTs that correlated the introduction of KD to an improvement of verbal receptive vocabulary and of reaction time in children affected by epilepsy, as well as an improvement in attention and memory in patients affected by multiple sclerosis. The results demonstrated causal evidence and stressed the need to increase the number of studies to demonstrate that 4:1 KD induces a cognitive improvement in neurological diseases.

The metabolomics techniques and the network-based integration methodologies will allow us to investigate the interaction among multiple genes, epigenetics and environmental factors, in order to better understand the pathogenesis of AD, to study and to monitor the activity and the efficacy of new therapeutic approaches such as KD and, finally, to develop a personalized management of the disease.

## Figures and Tables

**Table 1:** Clinical trials experimenting on the effectiveness of different types of ketogenic diets on Alzheimer’s cognitive impairment.

Authors	Objective	Subjects	Procedures	Results	Authors’ Comments
Reger et al. [23]	to explore, in individuals with memory disorders, whether hyperketonemia improves cognitive functioning (change from baseline in the Alzheimer’s disease (AD) Assessment Scale-Cognitive subscale, ADAS-Cog).	20 individuals, mean age 74.7 (S*.*D*.* = 6*.*7), with probable AD (*n* = 15; National Institute of Neurological and Communicative Disorders and Stroke and the Alzheimer's Disease and Related Disorders Association (NINCDS-ADRDA criteria; 9 = *ε*4+) or amnestic mild cognitive impairment (*n* = 5; 1 = *ε*4+); mildly to moderately cognitively impaired with a mean Mini-Mental State Examination (MMSE) of 22.0 (S*.*D*.* = 5*.*5).	double-blind placebo controlled design with two study visits; during each visit, subjects received one of two isocaloric conditions (690 calories) in a randomized order: emulsified medium chain triglycerides (MCTs), or emulsified long chain triglycerides as a placebo.	significant increases in levels of the ketone body β-hydroxybutyrate (β-OHB) were observed 90 min after treatment (*p* = 0*.*007) when cognitive tests were administered; MCT treatment facilitated performance on the ADAS-Cog for ε4− subjects, but not for ε4+ subjects (*p* = 0.04).	β-OHB elevations were moderated by apolipoprotein E (APOE) genotype (*p* = 0*.*036).
Henderson et al. [24].	to assess whether 90-day daily dosing of the oral ketogenic product AC-1202 (medium chain triglyceride composed of glycerin and caprylic acid, C8:0) improve cognitive performance; additional outcomes included how cognitive scores were influenced by APOE4 genotype status.	152 subjects diagnosed with mild to moderate AD according to NINCDS-ADRDA criteria and Diagnostic and Statistical Manual of mental disorders-IV (DSM-IV) criteria, with a MMSE score of between 14 and 24 (inclusive) at screen (86, age 76.9 ± 8.9 years, were allocated to AC-102, and 66, age 76.8 ± 7.4 years, to placebo).	AC-1202 was compared to Placebo in a randomized, double-blind, placebo-controlled, parallel-group study; subjects were on a normal diet and continued taking approved AD medications; pre- and post-dosing serum β-hydroxybutyrate (β-OHB) levels were evaluated. Cognitive performance change from baseline was assessed after 90 days by ADAS-Cog and AD Cooperative Study—Clinical Global Impression of Change (ADCS-CGIC).	AC-1202 rapidly elevated serum ketone bodies in AD patients and resulted in significant differences in ADAS-Cog scores compared to the Placebo after 45 and 90 days of treatment. Effects were most notable in APOE4(-) subjects who were dosage compliant.	adverse events were more frequently observed in participants receiving AC-1202 and concerned mainly transient, mild to moderate gastrointestinal effects; this medium-chain triglyceride preparation of fractionated coconut oil (caprylic trigyceride) has been approved for the treatment of AD in the USA.
Krikorian et al. [25]	the primary outcomes included measures of executive ability, long term memory, and mood obtained at pretreatment baseline and after the 6-week of the intervention: high carbohydrate or very low carbohydrate diet.	23 (10 men, 13 women) older adults with mild cognitive impairment (Clinical Dementia Rating, CDR), age 70.1 ± 6.2 years.	the subjects were randomly assigned to the 6-week dietary interventions consisted of high carbohydrate (50% of calories) and very low carbohydrate (5% to 10% of calories) diets, the latter intended to induce ketosis; all subjects also provided urine samples at the baseline and final visits for urinary ketone assessment; working memory and set switching aspects of executive ability was evaluated by The Trail Making Test part B, secondary or long term memory with the Verbal Paired Associate Learning and mood with the Geriatric Depression Scale; waist circumference, fasting serum glucose and insulin level were analyzed.	ketone levels were positively correlated with memory performance (*p* = 0.04); the primary finding indicated improved secondary memory performances for the low carbohydrate subjects; there was no effect of the intervention on the Trail Making Test part B and Geriatric Depression Scale; there were significant changes in anthropometric and metabolic values and in dietary parameters; after the intervention, weight, waist circumference, fasting glucose and insulin value were lower for the low carbohydrate but not high carbohydrate group.	these preliminary data provide evidence that dietary ketosis by means of carbohydrate restriction can provide neurocognitive benefit for older adults with early memory decline and increased risk for neurodegeneration. Correction of hyperinsulinemia and other mechanisms associated with ketosis such as reduced inflammation and enhanced energy metabolism also may have contributed to improved neurocognitive function; a prominent issue will be duration of the effects and whether there is persistence of benefit beyond the period of active intervention.
Rebello et al. [26]	to evaluated the effect of the daily consumption of an oil, composed of medium chain triglycerides (MCTs) for 24 weeks on serum ketone body concentrations (β-hydroxybutyrate [βHB]) and cognitive performance.	6 individuals ≥50 (58–78) years, with mild cognitive impairment (MCI).	pilot and feasibility, randomized double blind placebo-controlled parallel trial; participants received 56 g/day of either medium chain triglycerides (MCTs) or placebo for 24 weeks; serum β-hydroxybutyrate concentrations, apolipoprotein-E4 status, and cognitive assessments (Alzheimer's Disease Assessment Scale-Cognitive subscale (ADAS-Cog), Trail Making Test, and Digit Symbol Test) were carried out.	intake of MCT oil increased serum ketone bodies and improved memory, only in subjects with mild Alzheimer’s disease who did not have an APOE ε4 allele while intake of placebo did not show improvement in any of the cognitive measures tested.	Due to the small number of participants only the raw scores were examined. Consumption of 56 g/day of MCTs for 24weeks increases serum ketone concentrations and appears to be a candidate for larger randomized control trials in the future
Ota et al. [27]	to examine the effects of a single MCT supplemented ketogenic meal serving on cognition in elderly non-demented subjects	subjects were 19 non-demented elderly adults over 60 years old (13 females; mean age: 66.1 ± 2.9 years)	subjects underwent neurocognitive tests 90 and 180 min after oral intake of a ketogenic meal (Ketonformula^®^) containing 20 g of MCTs and an socaloric placebo meal without MCTs on separate days.	elevation of plasma ketone concentration after intake of a single ketogenic meal containing 20 g of MCTs was confirmed (all *p* < 0.001); as for cognition, improvements were observed in the digit span test, Trail-Making Test B, and the global score (Z = −2.4, *p* = 0.017) following the ketogenic meal and the change in the executive functioning score was positively correlated with that of the plasma β- hydroxybutyrate level; the cognition-enhancing effect was observed predominantly for individuals who had a relatively low global score at baseline (Z = −2.8, *p* = 0.005), compared to individuals with a high global score (Z = −0.7, *p* = 0.51).	plasma levels of ketone bodies were successfully increased after intake of the ketogenic meal; the ketogenic meal was suggested to have positive effects on working memory, visual attention, and task switching in non-demented elderly; the study is limited by the small sample size which may have resulted in false-positive and -negative results of the effect of the ketogenic meal in some cognitive tests.
Ohnuma et al. [28]	This clinical trial, carried out in Japan, analyzed the effect of 90-day administration of a ketogenic meal “Axona” (40 g of powder containing 20 g of caprylic triglycerides) on cognitive function in mild-to-moderate Alzheimer’s disease (AD) patients.	22 Japanese patients with sporadic AD at a mild-to-moderate stage (ten females, 12 males), mean age (± standard deviation) 63.9 (± 8.5) years, Mini-Mental State Examination (MMSE) score, 10–25, seven patients were ApoE4-positive.	prospective, open-label, observational study; Axona was administered for 3 months using an indurating, four-step dose-titration method (from 10 to 40 g per day) for 7 days before the trial, and examined the tolerance and adverse effects of this intervention; blood tests included: haemogram, alanine aminotransferase and aspartate aminotransferase, creatinine and urea nitrogen, glucose, glycohaemoglobin A1C, low-density lipoprotein and high-density lipoprotein, triacylglycerol, albumin, and total protein, and sodium, chloride, and potassium; serum total ketone bodies (acetoacetic acid and β-hydroxybutyric acid); the effect on cognitive function was assessed using the MMSE and Alzheimer’s Disease Assessment Scale (ADAS) cognitive subscale, Japanese version (ADAS-Jcog); *ApoE* genotypes were determined.	the tolerance of Axona was good, without severe gastrointestinal adverse effects; Axona did not improve cognitive function in our sample of AD patients, even in those patients without the ApoE4 allele; however, some ApoE4-negative patients with baseline MMSE score ≥14 showed improvement in their cognitive functions.	the modified dose-titration method, starting with a low dose of Axona, decreased gastrointestinal adverse effects in Japanese patients. Axona might be effective for some relatively mildly affected patients with AD (with cognitive function MMSE score of ≥14 and lacking the ApoE4 allele).
Taylor et al. [29]	The primary objective of Ketogenic Diet Retention and Feasibility Trial (KDRAFT) was to address the feasibility of implementing a very high-fat ketogenic diet (VHF-KD) intervention in AD participants; secondary objectives included evaluating the effects of a VHF-KD on cognition.	15 participants with AD. Individuals were eligible to the study if they had a clinical dementia rating (CDR) of very mild AD (CDR 0.5), mild AD (CDR 1), or moderate AD (CDR 2).	single-arm, pilot clinical study enrolled 15 participants with AD and required participants to maintain an MCT-supplemented KD for 3 months and then to resume a normal diet for 1 month (washout period). KD included 70% of energy as fat (including the MCT), 20% as protein, and carbohydrate less than 10% of energy; ketogenic ratio (lipid grams to non lipid grams) of 1:1 or better. MCT oil supplement (Now Foods, USA) contained a combination of C8:0 and C10:0 fatty acids; participants self-monitored urine ketones daily; serum electrolytes, renal function tests, liver function tests, and glucose levels were measured at the baseline and month 3 visits. Serum β-hydroxybutyrate (βHB) and insulin levels were measured at baseline, month 1, month 2, month 3, and washout, Homeostatic Model Assessment Index 2-Insulin Resistance (HOMA2-IR) values were calculated; Dual energy X-ray absorptiometry was used to assess body composition; MMSE, to identify cognitive impairment, and ADAS-cog, to measure changes in memory, language, praxis, and attention, were administered at baseline, at the end of the intervention (month 3), and after the 1-month washout.	7 CDR0.5, 4 CDR1, and 4 CDR2 participants were enrolled; 10 completers achieved ketosis; among the completers, the mean of the Alzheimer’s Disease Assessment Scale-cognitive subscale score improved by 4.1 points during the diet (P 5.02) and reverted to baseline after the washout. Only one participant’s ADAS-cog score declined while following the diet protocol. Serum βHB levels were significantly elevated at months 1, 2, and 3 compared with that of baseline and returned to the normal range at the end of thewashout period (0.12 mmol/L).	Because of small sample size and single-arm design, any interpretationp of this study’s cognitive performance data requires caution; the pilot trial justifies KD studies in mild Alzheimer’s disease.
